# Improving the Safety of *Staphylococcus aureus* Polyvalent Phages by Their Production on a *Staphylococcus xylosus* Strain

**DOI:** 10.1371/journal.pone.0102600

**Published:** 2014-07-25

**Authors:** Lynn El Haddad, Nour Ben Abdallah, Pier-Luc Plante, Jeannot Dumaresq, Ramaz Katsarava, Steve Labrie, Jacques Corbeil, Daniel St-Gelais, Sylvain Moineau

**Affiliations:** 1 Département de biochimie et de microbiologie, Faculté des sciences et de génie, Groupe de recherche en écologie buccale, Faculté de médecine dentaire, Félix d'Hérelle Reference Center for Bacterial Viruses, Université Laval, Québec, Canada; 2 Food Research and Development Centre, Agriculture and Agri-Food Canada, Saint-Hyacinthe, Québec, Canada; 3 Département de Médecine Moléculaire, Faculté de Médecine, Université Laval, Québec, Canada; 4 Département de Microbiologie et d'Infectiologie, Centre Hospitalier Affilié Universitaire Hôtel-Dieu de Lévis, Lévis, Québec, Canada; 5 Institute of Chemistry & Molecular Engineering, Agricultural University of Georgia, University Campus at Digomi, Tbilsi, Georgia; 6 Département des sciences des aliments et de nutrition, Faculté des sciences de l'agriculture et de l'alimentation, Dairy Science and Technology Research Centre/Institute of nutrition and functional foods, Université Laval, Québec, Canada; Centro Nacional de Biotecnologia – CSIC, Spain

## Abstract

Team1 (vB_SauM_Team1) is a polyvalent staphylococcal phage belonging to the *Myoviridae* family. Phage Team1 was propagated on a *Staphylococcus aureus* strain and a non-pathogenic *Staphylococcus xylosus* strain used in industrial meat fermentation. The two Team1 preparations were compared with respect to their microbiological and genomic properties. The burst sizes, latent periods, and host ranges of the two derivatives were identical as were their genome sequences. Phage Team1 has 140,903 bp of double stranded DNA encoding for 217 open reading frames and 4 tRNAs. Comparative genomic analysis revealed similarities to staphylococcal phages ISP (97%) and G1 (97%). The host range of Team1 was compared to the well-known polyvalent staphylococcal phages phi812 and K using a panel of 57 *S. aureus* strains collected from various sources. These bacterial strains were found to represent 18 sequence types (MLST) and 14 clonal complexes (eBURST). Altogether, the three phages propagated on *S. xylosus* lysed 52 out of 57 distinct strains of *S. aureus*. The identification of phage-insensitive strains underlines the importance of designing phage cocktails with broadly varying and overlapping host ranges. Taken altogether, our study suggests that some staphylococcal phages can be propagated on food-grade bacteria for biocontrol and safety purposes.

## Introduction

*Staphylococcus aureus* is one of the main causes of hospital associated infections [1] and foodborne contaminations [2]. Despite being found on the skin and in mucous membranes of healthy carriers, *S. aureus* is responsible for a wide range of diseases, including mild to severe skin infections, sepsis, endocarditis, and other life-threatening infections. Methicillin-resistant *S. aureus* (MRSA) are often found in a hospital or a community outbreak and their emergence is becoming a global concern [3]. In addition, some *S. aureus* strains cause food poisoning which results from the ingestion of staphylococcal enterotoxins secreted during growth in foods [2]. To combat the detrimental effects of staphylococcal growth, phages are being investigated as an alternative strategy.

However, certain staphylococcal phages encode virulence genes such as Panton-Valentine leukocidin, exfoliative toxin, and enterotoxin genes [4]–[8]. In fact, many temperate phages are a source of lysogenic conversion as they can integrate into the bacterial genome upon infection through the expression of lysogeny-related genes. When phages exist in a prophage state, an induction phenomenon can reactivate and excise their genome from the bacterial chromosome to initiate a lytic cycle. Temperate phages can ultimately play a role in the horizontal transfer of virulence factors from a donor host to a recipient cell [9]. Therefore, it is more suitable to use only virulent phages in a biocontrol application in order to ensure the absence of genes coding for unwanted traits.

To avoid the transfer of virulence genes, the use of a non-pathogenic bacterial strain or a “surrogate” to propagate these phages is preferable, as long as it does not induce changes in the characteristics of the phage. For example, the anti-*Listeria monocytogenes* phages are propagated on strains of non-pathogenic *Listeria innocua* [10]–[12]. Others showed that the propagation of polyvalent *Salmonella* phage phi PVP-SE1 on a non-pathogenic *Escherichia coli* strain did not change the microbiological properties and the DNA restriction profile of that phage. The availability of such non-pathogenic phage-production hosts will facilitate the purification process leading to a safer phage product [13].

Recently, the use of virulent staphylococcal phages as biocontrol agents has been further investigated prompted by the increasing emergence of strains resistant to antibiotics. Some polyvalent staphylococcal phages have been isolated and characterized in the last decade [14]–[19]. Of particular interest are the staphylococcal phages belonging to the *Myoviridae* family (dsDNA genome, icosahedral capsid, and contractile tail) known for their broad host range [20]–[23] and their ability to infect coagulase positive and negative staphylococci (CoPS and CoNS) of animal and human origins [22], [24].

In this study, a new polyvalent phage called Team1 (vB_SauM_Team1) belonging to the *Myoviridae* family was characterized following propagation on a CoPS strain of *S. aureus* and on a non-pathogenic CoNS strain of *Staphylococcus xylosus*. *S. xylosus* is a coagulase-negative staphylococcal species that has long been used as starter culture in Greek, Spanish, Italian and other traditional fermented sausage processes [25]. *S. xylosus* contributes to the aroma and color stability of the final product by minimizing rancidity and, in some cases, providing a bioprotective effect against microbial contamination [26], [27]. We also compared the microbiological and genomic properties of phage Team1 with those of two well-known polyvalent staphylococcal phages K and 812. In parallel, we genotyped a wide range of *S. aureus* strains isolated from different sources and verified the polyvalent nature of the three phages.

## Materials and Methods

### Bacterial strains and media

The CoPS strain *S. aureus* SA812 and CoNS strain *S. xylosus* SMQ-121 were used to propagate the phages (Table 1). *S. xylosus* SMQ-121 is a commercially available meat starter. The strains were obtained from the Félix d'Hérelle Reference Center for Bacterial Viruses (www.phage.ulaval/ca/). We established the host ranges of the phages using fifty-six additional *S. aureus* strains isolated from different sources. Tryptic Soy Broth (TSB) was used for culture of all staphylococcal cells.

**Table 1:** Genotyping of the 57 *S. aureus* strains used in this study.

Strains	Source of isolation	ST (CC)	References
SA812	Unknown (Czech Republic)	ST30 (CC30)	[23]
HER1101	Unknown	ST707 (CC47)	This study
HER1049	Unknown	ST25 (CC25)	
HER1225	Unknown	ST9 (CC9)	
A170	Infected wounds (Italy)	ST45 (CC45)	[19]
SMQ1281, SMQ1282	Raw cheese samples (MAPAQ, Canada)	ST352 (CC97)	This study
SMQ1283 to SMQ1299	Mastitis infections (CBMRN, Canada)	ST352 (CC97)	
SMQ1300, SMQ1301	Mastitis infections (CBMRN, Canada)	ST2187 (CC97)	
SMQ1302 to SMQ1319	Mastitis infections (CBMRN, Canada)	ST151 (CC151)	
SMQ1320	Mastitis infections (CBMRN, Canada)	ST351 (CC151)	
SMQ1321	Mastitis infections (CBMRN, Canada)	ST126 (CC126)	
SMQ1322	Mastitis infections (CBMRN, Canada)	ST2270 (CC126)	
CMRSA1	Health-care associated (HA)	ST45 (CC45)	[3]
CMRSA2	Health-care associated (HA)	ST5 (CC5)	
CMRSA3	Health-care associated (HA)	ST241 (CC8)	
CMRSA4	Health-care associated (HA)	ST36 (CC36)	
CMRSA5	Health-care associated (HA)	ST8 (CC8)	
CMRSA6	Health-care associated (HA)	ST239 (CC239)	
CMRSA7	Community acquired (CA)	ST1 (CC1)	
CMRSA8	Health-care associated (HA)	ST217 (CC22)	
CMRSA9	Health-care associated (HA)	ST8 (CC8)	
CMRSA10	Community acquired (CA)	ST8 (CC8)	

### Bacterial strain identification and genotyping

The staphylococcal species of all strains was first confirmed through 16S analysis and the API Staph kit (BioMérieux™). We amplified the 16S ribosomal RNA gene by extracting the bacterial genomic DNA [28] and performing a Polymerase Chain Reaction using universal primers 27 forward and 1492 reverse, carried out with a PTC-200 machine (MJ Research, Peltier thermal cycler) as follows: 1 hold at 94°C for 3 min then 35 cycles of 94°C for 1 min, 58°C for 1 min, and 73°C for 1 min 45 s. A final elongation step at 73°C for 5 min was performed after the final cycle. The PCR products were sequenced at the genomic platform of the Centre Hospitalier de l'Université Laval using an ABI Prism 3100 apparatus (Applied Biosystems, Foster City, CA). DNA sequences were compared with sequences available in the GenBank database from the National Center of Biotechnology Information (NCBI) using BLAST analysis (http://www.ncbi.nlm.nih.gov/BLAST/) as well as the Ribosomal Database Project [29]. Furthermore, we genotyped all S. aureus strains using the MLST method [28]. This method targets the sequencing of seven housekeeping genes of ∼450 bp. The PCR reactions consisted of: 5-min denaturation at 95°C, followed by 30 cycles of annealing at 55°C for 1 min, extension at 72°C for 1 min, and denaturation at 95°C for 1 min, followed by a final extension step at 72°C for 5 min and sequencing of the PCR products. Each gene sequence obtained was given an appropriate allele number. The combination of the 7 alleles defined the allelic profile, which corresponded to its Sequence Type (ST). This was identified using the MLST database website [28]. Moreover, the different STs acquired were clustered into clonal complexes (CC) using the eBURST algorithm. This algorithm is programmed to assemble the STs that have 6 or 7 alleles in common under the same CC [30].

### Phage preparations

Phage Team1 was isolated as a virulent phage in the Republic of Georgia following a hospital staphylococcal infection [31], [32]. Phage Team1 was propagated for four passages in TSB medium at 37°C on its host strain *S. aureus* SA812 (i.e., Team1-SA812) as well as on *S. xylosus* SMQ-121 (i.e., Team1-SMQ121). Two well-known polyvalent staphylococci phages, phi812 [23] and K [22] were also propagated on both of these strains. The phage preparations were named 812-SA812, 812-SMQ121, K-SA812, and K-SMQ121 for phages phi812 and K, respectively. To propagate the phages, bacteria were grown at 37°C to an optical density at 600 nm of 0.1, and then approximately 10^5^ phages were added to the medium. Incubation continued at 37°C until complete bacterial lysis, and the resulting lysate was filtered using a 0.45-µm syringe filter.

### Electron microscopy

A 1.5-ml sample of phage lysate (titer of at least 10^9^ PFU/ml) was centrifuged at 23,500×g for 1 h at 4°C. The supernatant was removed, leaving approximately 100 µl in the tube. The phage pellet was washed twice with 1.5 ml of ammonium acetate (0.1 M, pH 7.5). The residual volume (100 µl) was used to prepare the observation grid as follows: 10 µl of the staining solution (2% phosphotungstic acid, pH 7.0) was deposited on a Formvar carbon-coated grid (200 mesh; Pelco International). After 30 s, 10 µl of the washed lysate was mixed with the stain by pipetting up and down. After 90 s, the residual liquid was removed from the grid by touching the edge with blotting paper. Phages were observed at 80 kV using a JEOL 1230 transmission electron microscope available at the Institut de Biologie Intégrative et des Systèmes (IBIS) of the Université Laval.

### Microbiological assays

The one-step growth curve assays of Team1-SA812 and Team1-SMQ121 were carried out in triplicate as previously described [33] with a multiplicity of infection (MOI) of 0.05 and at a temperature of 37°C. The burst size was calculated by dividing the average phage titer after the exponential phase by the average titer before the infected cells began to release virions [33]. Phage counts of Team1, phi812, and K propagated on both species, expressed as EOP values, were obtained using a double-layer plaque titration method. In brief, 5 µl of a phage preparation (undiluted lysate and serially diluted (10^−1^ to 10^−8^)) were spotted on TSB soft agar containing 100 to 200 µl of a staphylococcal culture previously grown overnight. Two biological and two technical repetitions were done for each phage and strain. The EOP values were calculated by dividing the titer of the phage on the tested strain by the phage titer on its host strain. In total, 58 strains (57 *S. aureus* and 1 *S. xylosus*) were tested against the three phages and their derivatives.

### Phage DNA preparation and sequencing

Phage Team1-SA812, Team1-SMQ121, 812-SA812, 812-SMQ121, K-SA812, and K-SMQ121 genomic DNAs were isolated using a Lambda Maxi kit (Qiagen) with modifications reported elsewhere [34]. The EcoRV (Roche Diagnostics) restriction profile of the six phages were compared to confirm differences between phages Team1, phi812, and K, as well as identities with their respective derivatives amplified on the other strain. The DNA fragments were separated in a 0.8% agarose gel, stained with EZVision (Amresco), and photographed under UV illumination. The sequencing was performed using pyrosequencing technology on a 454 FLX instrument available at the Plateforme d'analyses génomiques of the IBIS. Reads were assembled into a single contig with 32-fold coverage for Team1-SMQ121 and 86-fold coverage for Team1-SA812. For phages phi812-SA812 and phi812-SMQ121, reads were assembled into a single contig with 32-fold and 71-fold coverage, respectively. Finally, for phages K-SA812 and K-SMQ121, the coverage was 72-fold and 62-fold coverage, respectively.

### Bioinformatic analysis

The genomes were analyzed using Staden [35] and BioEdit 7.0.9.0 [36]. Open reading frames (ORF) were identified using the ORFinder tool [37] and GeneMarkS [38]. Each ORF begins with a starting codon (AUG, UUG or GUG) and most are preceded by a Shine-Dalgarno sequence specific to staphylococci and optimally placed approximately 10 nucleotides upstream of the start codon [39]. The putative function of each protein was deduced by homology searches using blast2GO [40]. The theoretical isoelectric point (pI) and the molecular mass (MM) of each deduced phage protein was obtained using Compute pI/Mw available on the ExPASy Web page (http://ca.expasy.org/tools/pi_tool.html). Finally, tRNAs were identified using the tRNAscan-SE server [41] and the ARAGORN program [42]. The genome of phage Team1 was compared with the database on NCBI using BLAST. The two phages sharing the highest nucleotide similarity with phage Team1 were selected. The genomes of these three phages were compared at the DNA level and results were presented in circular alignments using Circos software [24]. Codon usage was determined using the codon usage tool accessible through the DNA 2.0 Web server (DNA 2.0, Menlo Park, CA) and the Countcodon program available on the Kazusa DNA Research Institute Web page (http://www.kazusa.or.jp/codon/). The percentages of synonymous codon usage were calculated for each amino acid. The bacterial codon usage of *S. aureus* JH1 was obtained from the Kazusa DNA Research Institute database for comparison purposes.

### Nucleotide sequence accession number

The genome sequence of *S*. *xylosus* Team1-SMQ121 phage was deposited in GenBank under accession number KC012913.

## Results and Discussion

### Growth curves comparison of phages Team1-SA812 and Team1-SMQ121

**Figure 1:**
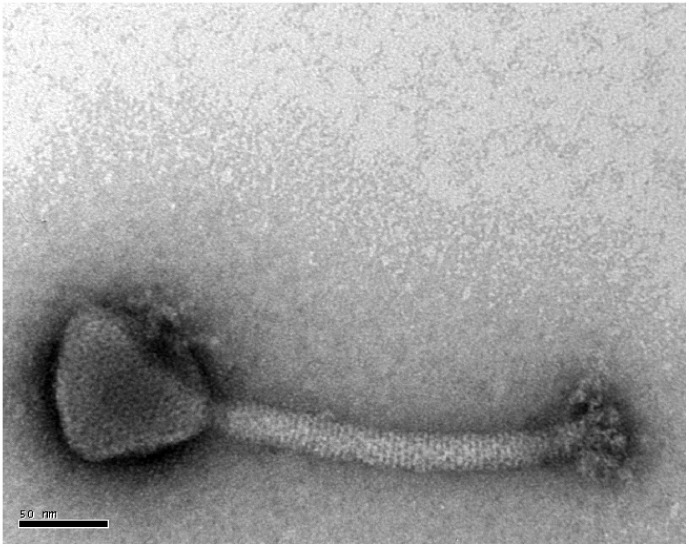
Electron micrograph of phage Team1. The bar scale  = 50 nm.

Team1 has an icosahedral capsid with a diameter of 90.4±4.1 nm and a long contractile sheath 227.2±7.6 nm in length and 21.6±1.5 nm in width, a morphology resembling other staphylococcal *Myoviridae* [21], [23] (Fig. 1). Before propagating Team1 on *S. xylosus*, the species identity was confirmed using 16S rRNA analysis and API-Staph kit detection. Strain SMQ-121 had 99% identity with *S. xylosus* strains through 16S rRNA analysis and 96.7% identity using the API-Staph kit, confirming that this strain belongs to the *S. xylosus* species.

**Figure 2:**
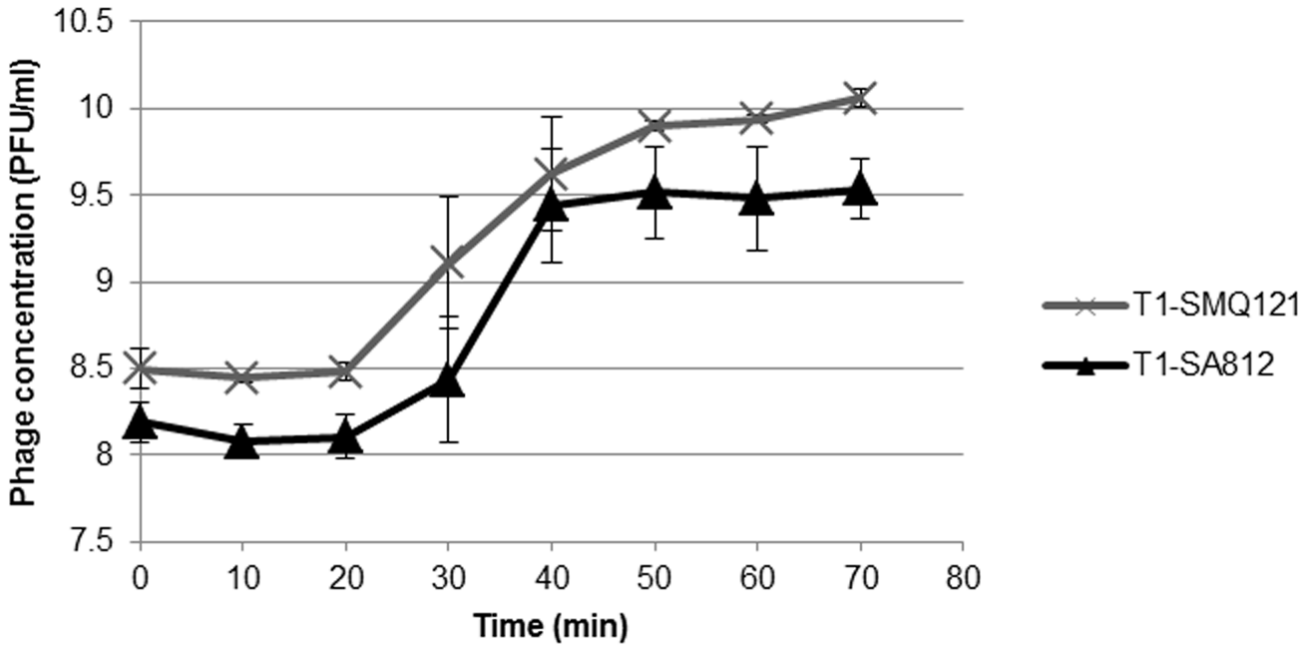
One-step growth curves of both Team1-SA812 (▴) and Team1-SMQ121 (×). Error bars indicate the standard deviation for three trials. The first phage count (time zero) occurred approximately 10 min after adding the phage to the cells [33].

The impact of propagating phage Team1 on both *S. aureus* and *S. xylosus* species was determined by comparing their respective growth curves (Fig. 2). The burst size of Team1 was similar on both hosts, with 31±7 PFU per infected *S. aureus* SA812 cell and 37.5±3 pfu per infected *S. xylosus* SMQ-121 cell after approximately 35 minutes. The latency periods (defined as the time interval between the absorption and the beginning of the first burst) were also identical at approximately 20 minutes when grown at 37°C. These values are similar to other *Myoviridae* staphylococcal phages (20, 21, 23). The myophage phi812 that has a longer latent phase and a lower burst size [23] and the newly isolated phages SAH-1 and Stau2 that have similar latent periods but larger burst sizes of 100 pfu per infected cell [20], [21].

### Genome analyses

Phages Team1-SA812 and Team1-SMQ121 DNA were extracted, sequenced, and compared. The complete genome of Team1 is a 140,903 bp, linear double-stranded DNA. In addition, the Team1 genome has a GC content of 30.3%, which is lower than the estimated GC contents of *S. aureus* and *S. xylosus*, which have GC contents of 32.8% and 34.2%, respectively [25], [43], [44]. However, this GC content is similar to other staphylococcal *Myoviridae* phages [45]. The ends of the phage Team1 genome contain 8,053 bp of long direct terminal repeats (LTRs) separated from the non-redundant part of the virion DNA by 12 bp inverted repeat sequences 5′-TAAGTACCTGGG- 3′ and 5′-CCCAGGTACTTA-3′, which are characteristic features of Twort-like viruses [45]. Homologous recombination between the LTRs permits circularization of infecting phage DNA. Phage Team1 encodes 217 predicted ORFs, 44 of which have a putative function (20%) (Table S1). As with most phages, the Team1 genome can be divided into several modules, including two DNA replication modules and two lysis modules separated by DNA packaging and morphogenesis modules. This modular organization is common among phages belonging to the *Myoviridae* family [8]. The Team1 genome also encodes four tRNAs. The tRNA^Met^ (ATG) is located between ORF33 and ORF34, whereas the three others (tRNA^Trp^ - TGG, tRNA^Phe^ - TTC and tRNA^Asp^ - GAC) are located between ORF76 and ORF77 in proximity to a lysis module. No known virulence factors were found in the phage Team1 genome establishing its safety. Moreover, no lysogenic module or known integrase-related genes were found in its genome, confirming its strictly virulent nature. Finally, comparative analysis of restriction profiles (EcoRV) and genome sequences indicated 100% identity between phages Team1-SA812 and Team1-SMQ121, showing that propagating this phage on two distinct staphylococcal species did not lead to genomic changes.

### Comparative genomics

**Figure 3:**
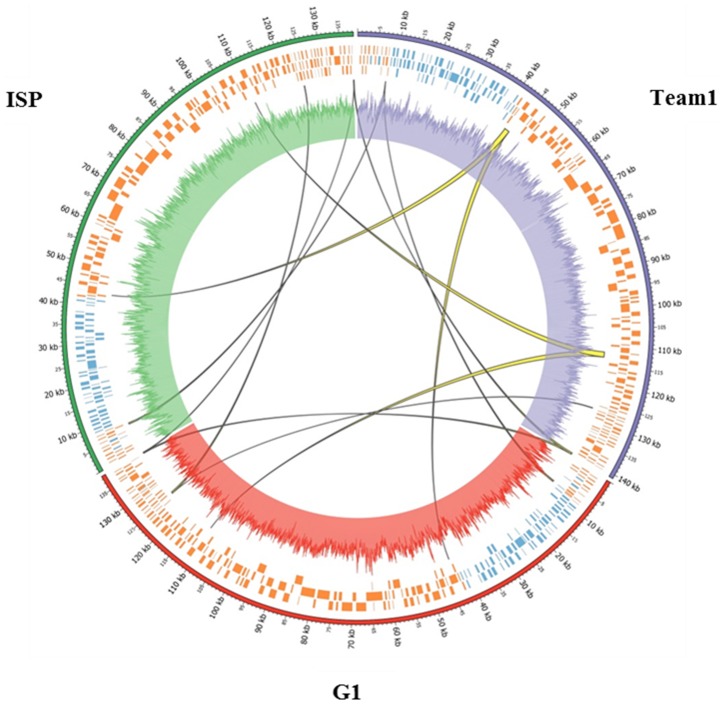
Circular genome comparison of phages Team1, G1, and ISP. Phages Team1, G1, and ISP genomes and GC content are represented, respectively, using the colors purple (upper right), red (center), and green (upper left). For each phage genome, coding regions in minus (blue) and plus (orange) strands are shown in their respective frames. Yellow lines show the additional acquired sequences between these three genomes. The width of the line depends on the length of the acquired sequences [24].

The genome of phage Team1 was found to be 97% identical to the published genomes of staphylococcal phages ISP [18] as well as G1 [39]. In fact, most staphylococcal myophages, except phage Twort, share identity at the DNA level between each other, ranging from 88.3% to 99.9% [45]. When comparing phage Team1 with G1 and ISP genomes, deletions of 375 bp and 379 bp were observed in the Team1 genome at positions 7,535 and 39,917 respectively (Fig. 3). Deletions were flanked by direct repeat sequences, suggesting that the deletions may have occurred through intramolecular recombination between direct repeats of homologous DNA. The second deletion corresponded to non-coding regions. However, the 375-bp deletion led to the removal of a gene coding for a putative DNA terminal protein, which may explain the shorter LTR sequence in Team1 genome as compared to that of phages G1 and ISP [45]. Insertions of 1,348 bp, 1,375 bp, 146 bp (compared to phage G1), and 260 bp (compared to phage ISP) were also identified at positions 42,626,111,668, and 140,903 in Team1 genome [24]. Two genes were added with these insertions, *orf86* and *orf155*. The former is a hypothetical gene whereas *orf155* encodes a putative intron-encoded endonuclease [46]. The last insertions constituted repetitive non coding sequences.

The three phages possess the same four tRNAs including two matching the unique codons for methionine and tryptophan. We also investigated phage codon usage [47] and compared it to *S. aureus* codon usage obtained from the Countcodon program. The codon usage is highly similar between the three phages. While the four tRNAs encoded by the phages are similar to the host tRNAs, significant differences in codon usage were noted between these myophages and the *S. aureus* strain analyzed (Table 2), which likely favors specific phage protein production [48].

**Table 2:** Codon usage of *S. aureus* JH1 and phages G1 and ISP for the amino acids encoded by the Team1 tRNAs (in bold).

Amino-acid	Codon	Team1	G1	ISP	*S. aureus* JH1
Cys	TGT	74.0	67.0	66.0	80.6
	TGC	26.0	33.0	34.0	19.4
**Asp**	**GAC**	**28.0**	**28.0**	**27.0**	**22.0**
Glu	GAG	34.0	32.0	31.0	16.4
**Phe**	**TTC**	**30.0**	**32.0**	**34.0**	**27.2**
Gly	GGC	7.0	8.0	9.0	15.4
Ile	ATA	46.0	41.0	41.0	22.0
	ATT	39.0	43.0	42.0	60.6
Lys	AAG	38.0	37.0	36.0	19.0
Leu	TTA	40.0	37.0	38.0	59.0
	CTA	19.0	18.0	17.0	9.5
**Met**	**ATG**	**100.0**	**100.0**	**100.0**	**100.0**
Pro	CCA	28.0	32.0	31.0	50.6
Gln	CAA	62.0	64.0	63.0	87.9
Arg	AGG	24.0	26.0	27.0	4.3
	AGA	59.0	57.0	58.0	33.7
	CGT	7.0	7.0	5.0	37.7
Thr	ACC	18.0	20.0	21.0	4.6
**Trp**	**TGG**	**100.0**	**100.0**	**100.0**	**100.0**
End	TAA	49.0	51.0	50.0	73.5

### Genotyping of *S. aureus* strains

In the next step, we determined the host range of phages Team1-SA812 and Team1-SMQ121. Before comparing the host ranges of these phages, we collected a panel of 57 *S. aureus* from various sources such as raw cheese, mastitis infections, clinical and hospital associated infections, and community acquired infections. We typed these hosts to assess the diversity of this bacterial set of strains. The typing was done using the MLST method, assigning each strain an ST number, and clustering them into CCs using the eBURST algorithm (Table 1).

The genotypes of the strains differed according to the origin of isolation. *S. aureus* strains isolated from raw cheese or from mastitis infections were found to share some similarities in their CC groups. *S. aureus* is the most common cause of bovine mastitis and often found in raw milk [49]. Mastitis is characterized by the inflammation of mammary glands inducing bacterial infections that result in global economic losses to the dairy industry. The studied *S. aureus* strains isolated from milk could be divided into six groups according to their ST numbers (352, 151, 351, 2270, 2187, and 126). However, when applying the eBURST algorithm and assembling STs sharing at least 6 alleles out of 7, the *S. aureus* strains isolated from milk were divided into three groups or three CCs, i.e., CC97, CC126, and, CC151. The majority of these strains belongs to CC97 (47.5%) and CC151 (47.5%). Other studies showed that the latter CCs are distributed worldwide and constitute the major groups causing mastitis infection outbreaks, mainly in North and South America and Europe [50], [51]. However, CC126 is most predominant in Brazil, along with CC97 [52]. Unlike CC97, CC151 and CC126 are exclusively confirmed in bovine isolates. It is also hypothesized that bovine strains worldwide derived from CC97. The CC97 lineage has existed for more than 50 years and emerged earlier than CC151 [50].

The other strains, whether MSSA (methicillin-sensitive *S. aureus*) or MRSA, had different STs and CCs, demonstrating source-dependent genotypes. The MSSA strains, HER1101, HER1049, HER1225, A170, and the host strain SA812, belong to CC707, CC25, CC9, CC45, and CC30 respectively. Conversely, the Canadian MRSA (CMRSA), hospital and community-acquired, have their own separate clustering and belong to different clonal complexes, i.e., CC1, CC5, CC8, CC22, CC36, CC45, CC239. Two clonal complexes share some similarities between the MSSA and MRSA strains studied. Strains CMRSA1 and A170 belong to CC45. Furthermore, CC30 (*S. aureus* SA812) and CC36 (CMRSA4 relevant to EMRSA-16 and USA200) have only two loci of difference in their allelic profile. Additionally, virulent MRSA strains belonging to CC1, CC5, and CC8 are responsible for several outbreaks in North America [3], [53] and are shifting to animal hosts as well [54].

### Host range comparisons of phages

After typing the 57 *S. aureus* strains, the host ranges of phages Team1-SA812 and Team1-SMQ121 were examined. We also tested the host ranges of the known polyvalent staphylococcal phages phi812 and K to compare with Team1. These two phages were also amplified on *S. aureus* SA812 and *S. xylosus* SMQ-121 and their genome sequenced to confirm their identity. No mutation was found when amplified on both hosts, as observed for Team1 (data not shown).

The results of the host ranges of the wild-type phages Team1, phi812, and K propagated on both *S. aureus* SA812 and *S. xylosus* SMQ-121 are reported in Table 3. Each of the three phages propagated on *S. aureus* SA812 infected 52 of the 57 *S. aureus* tested. In fact, 56 out of the 57 strains could be infected by at least one myophage. Only strain 1049 (ST25, CC25) was resistant to all three myophages. Of note, this strain is sensitive to *Podoviridae* phages P68 and 44AHJD (data not shown). When the same phages were propagated on *S. xylosus* SMQ-121, phages Team1 and phi812 infected 53 strains, while phage K infected 51 strains. Overall, 55 out of the 57 strains could be infected by at least one myophage propagated on *S. xylosus* SMQ-121. Only the phage sensitivity of strain SMQ1321 (ST126, CC126) was affected by the propagating host, suggesting the presence of host factors that modify the phage behavior in this unique case [48].

**Table 3:** Host range of phages Team1, phi812, and K propagated on *S. aureus* SA812 and on *S. xylosus* SMQ-121.

	*S. aureus* SA812				*S. xylosus* SMQ-121		
		Team1	phi812	K	Team1	phi812	K
	**SA812**	1.0	1.0	1.0	1.0	1.6	6.7
	**SMQ-121**	0.2	1.6	1.3	1.0	1.0	1.0
**Other**	**1101**	1.0	0.3	0.3	0.3	0.2	0.3
**strains**	**1049**	0	0	0	0	0	0
	**1225**	0.9	0.1	9.4E-2	0.5	0.3	0.7
	**A170**	1.4	3.8E-2	0	5.7	1.9E-2	0.7
**Raw**	**SMQ1281**	2.5	3.2	1.4	1.0	0.3	1.2
**milk**	**SMQ1282**	2.8	4.5	1.9	6.1	0.4	0.5
	**SMQ1283**	1.3	1.1	1.5	2.8	3.6	6.7
	**SMQ1284**	2.3	1.8	2.1	1.6	4.0	10.0
	**SMQ1285**	1.4	1.6	1.7	2.6	2.7	13.0
	**SMQ1286**	1.4	2.7	1.8	1.6	2.7E-2	3.3E-3
	**SMQ1287**	1.0	0.7	1.9	1.2	5.7	9.2
	**SMQ1288**	1.3	1.3	1.6	0.7	3.7	5.0
	**SMQ1289**	1.5	2.2	1.5	0.9	1.3	8.3
	**SMQ1290**	1.2	2.0	1.5	1.9	3.6	11.0
	**SMQ1291**	1.5	1.8	1.4	2.6	6.0	7.5
	**SMQ1292**	7.7E-7	1.1E-5	6.0E-6	2.3E-4	4.8E-5	0
	**SMQ1293**	1.9	1.6	1.7	2.8	8.4	11.0
	**SMQ1294**	1.7	0.9	1.0	1.9	6.0	6.7
	**SMQ1295**	1.3	1.3	0.9	2.0	0.4	1.1
	**SMQ1296**	2.4	3.1	4.1	1.3	1.2	1.6
	**SMQ1297**	2.5	1.3	1.0	0.9	0.9	3.8
	**SMQ1298**	0.3	1.5	0.7	3.0	0.7	0.2
	**SMQ1299**	0.5	2.3	1.8	1.5	0.6	1.3
	**SMQ1300**	0.5	1.7	0.8	1.5	0.3	0.9
**Mastitis**	**SMQ1301**	0.2	1.8	0.2	2.0	0.6	0.5
	**SMQ1302**	4.0	2.3	0.4	0.9	0.8	1.2
	**SMQ1303**	0.5	2.0	1.4	1.7	0.5	3.3
	**SMQ1304**	2.0	1.6	2.9	1.9	7.2	9.2
	**SMQ1305**	1.7	1.6	1.4	2.1	8.4	7.5
	**SMQ1306**	2.4	2.9	2.0	2.3	3.3	11.0
	**SMQ1307**	0	0.4	0.6	0	0.5	0.7
	**SMQ1308**	2.0	1.8	1.6	1.9	2.5	18.0
	**SMQ1309**	2.4	3.7	5.9	1.4	2.2E+2	41.0
	**SMQ1310**	1.6	3.1	0.4	1.4	6.1	19.0
	**SMQ1311**	1.9	2.7	1.6	2.1	1.9	14.0
	**SMQ1312**	2.4	2.7	1.5	3.0	4.8	10.0
	**SMQ1313**	1.6	2.5	1.3	3.5	6.0	6.7
	**SMQ1314**	0.8	1.6	0.9	2.8	12.0	7.5
	**SMQ1315**	0.8	2.5	1.4	1.4	9.6	5.0
	**SMQ1316**	1.1	2.7	1.5	1.4	13.0	11.0
	**SMQ1317**	1.5	1.5	1.4	2.6	12.0	9.2
	**SMQ1318**	1.5	3.8	1.9	2.4	0.7	0.4
	**SMQ1319**	0.1	2.5	2.2	2.0	1.0	0.7
	**SMQ1320**	2.5	8.2	4.4	2.8	0.9	1.9
	**SMQ1321**	1.9E-5	3.6E-6	2.2E-6	0	0	0
	**SMQ1322**	1.5E-4	4.0E-4	6.0E-5	1.4E-3	1.4E-4	0
	**MRSA1**	1.6	0.1	3.8	1.4	7.5E-2	1.3E-5
	**MRSA2**	0	1.2	0.8	0	0.2	0.4
	**MRSA3**	1.9	2.7E-2	3.1E-7	1.6	4.5E-3	1.3E-5
	**MRSA4**	2.2	1.1	1.0	1.4	1.1	0.5
**MRSA**	**MRSA5**	1.8	0	0	0.9	0	0
	**MRSA6**	0.9	0	0	6.9	0	0
	**MRSA7**	6.3E-2	0.3	0.3	0.1	0.1	0.1
	**MRSA8**	3.8	1.0	3.4E-6	0.2	0.2	1.9E-4
	**MRSA9**	0.9	4.2	1.5	1.1	0.4	0.5
	**MRSA10**	4.4E-2	2.0E-2	5.0E-3	1.8	8.5E-3	6.2E-2

The results are expressed as EOP values. Bold characters indicate host strain.

0 indicates that no plaque was observed with high titer phage preparations.

While the general host ranges remained unchanged, the level of sensitivity as determined by EOP values varied in only a few cases. For example, the three phages had a reduced EOP (<10^−4^) when plated on *S. aureus* SMQ1292 (ST352, CC97), SMQ1321, and SMQ1322 (ST2270, CC126). Similarly, phage K propagated on *S. xylosus* had reduced EOP values (10^−3^ to 10^−5^) when plated on SMQ1286, CMRSA1, CMRSA3 and CMRSA8 strains. The EOP of phage K propagated on *S. aureus* SA812 dropped also to 10^−7^ and 10^−6^ when plated on CMRSA3 and CMRSA8 strains, respectively (Table 3). A similar phage K behavior was observed in another study [22].

Taken altogether, the host ranges and EOP values were mostly similar when comparing phages amplified on both hosts, indicating that *S. xylosus* SMQ-121 is a suitable host to produce these myophages. Although the results demonstrate the polyvalent nature of the three phages tested, at least one *S. aureus* strain was resistant to these polyvalent phages, illustrating the importance of using phage cocktails to cover a wider range of strains. It also suggests that such strain should be used to uncover novel phages.

In conclusion, a new polyvalent staphylococcal phage was characterized. We found that phage Team1, as well as the well-known phages K and phi812, can also be propagated on a non-pathogenic *S. xylosus* strain and be highly effective in killing a large panel of *S. aureus* strains obtained from various sources and belonging to different genotypes. This study proposes the use of food-grade bacteria to amplify virulent and polyvalent staphylococcal phages in order to increase the safety of these promising biocontrol agents for both medical and food applications.

## Supporting Information

Table S1 **ORF identification, putative function, and comparison of Team1 genome with sequences available in public databases.**
(DOCX)
