# Supplementary material for: Improving the Safety of Staphylococcus aureus Polyvalent Phages by Their Production on a Staphylococcus xylosus Strain
Source: PLoS One. 2014 Jul 25;9(7):e102600. doi: 10.1371/journal.pone.0102600 (PMC4111496; doi:10.1371/journal.pone.0102600)
Supplement: Table S1 — ORF identification, putative function, and comparison of Team1 genome with sequences available in public databases. (DOCX) [file pone.0102600.s001.docx]

**Table S1. ORF identiﬁcation, putative function, and comparison of Team1 genome with sequences available in public databases.**

| ORF | Start | End | Length  (a.a.) | pI/Mw  (KDa) | SD sequence 5' - AGGAGG -3' | Putative function of the deduced protein | Best hit with BLAST | Number of  identical a.a./total  number of a.a.(%) | Length (a.a.) | E-value | Accession number (GenBank) |
| --- | --- | --- | --- | --- | --- | --- | --- | --- | --- | --- | --- |
| 1 | 496 | 795 | 99 | 4.4/11.6 | AGGAGAtgaaatag**ATG** |  | G1, ORF150 | 99/99 (100) | 99 | 4.0E-64 | [YP_241022.1](http://www.ncbi.nlm.nih.gov/protein/66395090?report=genbank&log$=prottop&blast_rank=1&RID=07PAJCRK014) |
| 2 | 811 | 996 | 61 | 5.7/6.8 | AGGAGGataagtaatc**ATG** |  | G1, ORF231 | 61/61 (100) | 61 | 3.0E-30 | YP_241023.1 |
| 3 | 1103 | 1393 | 96 | 4.1/11.3 | AGGAGAgatata**ATG** |  | G1, ORF156 | 96/96 (100) | 96 | 1.0E-60 | YP_241024.1 |
| 4 | 1393 | 1680 | 95 | 7.6/10.9 | AGGAGAtgttata**ATG** |  | G1, ORF158 | 95/95 (100) | 95 | 2.0E-60 | YP_241025.1 |
| 5 | 1680 | 1973 | 97 | 4.3/11.5 | AGGAGAgattata**ATG** |  | G1, ORF154 | 97/97 (100) | 97 | 3.0E-63 | YP_241026.1 |
| 6 | 1977 | 2234 | 85 | 6.1/10.2 | AGGAGAtgtaacta**ATG** |  | G1, ORF175 | 85/85 (100) | 85 | 3.0E-54 | YP_241027.1 |
| 7 | 2312 | 2551 | 79 | 6.0/9.2 | AGGGGGaagttagg**ATG** |  | G1, ORF183 | 79/79 (100) | 79 | 1.0E-49 | YP_241028.1 |
| 8 | 2562 | 2909 | 115 | 4.5/13.7 | AGGAGGgatagg**ATG** |  | K, ORF118 | 115/115 (100) | 115 | 2.0E-76 | YP_024546.1 |
| 9 | 3458 | 3120 | 112 | 4.5/13.5 | AAAAGGatttgatttaa**ATG** |  | G1, ORF128 | 112/112 (100) | 112 | 1.0E-70 | YP_241030.1 |
| 10 | 3769 | 4077 | 102 | 4.7/11.8 | TGGAGAtgatttaa**ATG** |  | G1, ORF145 | 102/102 (100) | 102 | 1.0E-68 | YP_241031.1 |
| 11 | 4283 | 4570 | 95 | 5.2/11.0 | AGGATTtgattatt**ATG** |  | GH15, ORF003 | 92/95 (97) | 95 | 5.0E-61 | YP_007002126.1 |
| 12 | 4620 | 4811 | 63 | 9.8/7.7 | AGGATAtgataat**ATG** |  | G1, ORF221 | 63/63 (100) | 63 | 5.0E-37 | YP_241033.1 |
| 13 | 5615 | 5127 | 162 | 9.7/19.6 | AGGAGGagtaaaaaa**ATG** | Endonuclease | G1, ORF085 | 162/162 (100) | 162 | 7.0E-114 | YP_241035.1 |
| 14 | 5783 | 5941 | 52 | 10.4/6.1 | AGGGATtgaacaaa**ATG** |  | ISP,ORF144 | 52/52 (100) | 52 | 1.0E-26 | CCA65876.1 |
| 15 | 6011 | 6142 | 43 | 10.0/5.1 | AGGAAAtgataaaa**ATG** |  | G1, ORF297 | 43/43 (100) | 43 | 5.0E-21 | YP_241036.1 |
| 16 | 6309 | 6632 | 107 | 4.8/12.4 | AGGAATtgattata**ATG** |  | ISP, ORF146 | 107/107 (100) | 107 | 3.0E-71 | CCA65878.1 |
| 17 | 6732 | 6968 | 78 | 4.2/9.1 | AGGAATtgatacaa**ATG** |  | A5W, ORF194 | 78/78 (100) | 78 | 9.0E-47 | ACB89187.1 |
| 18 | 7048 | 7518 | 156 | 3.6/17.8 | AGGATTtgattatt**ATG** |  | A5W, ORF001 | 156/159 (98) | 159 | 4.0E-104 | ACB88992.1 |
| 19 | 7548 | 7673 | 41 | 4.1/4.6 | AAGCGGaacaatacag**ATG** |  | G1, ORF166 | 41/89 (46) | 89 | 5.0E-20 | YP_241041.1 |
| 20 | 7758 | 7940 | 60 | 5.4/7.4 | AGGAAAtgatgaac**ATG** |  | SA5, ORF151 | 60/62 (97) | 62 | 6.0E-33 | AFV80806.1 |
| 21 | 8506 | 8270 | 78 | 4.8/9.6 | AGGAGAataagtaat**ATG** |  | SA5, ORF152 | 78/240 (32.5) | 240 | 2.0E-49 | AFV80807.1 |
| 22 | 8993 | 8508 | 161 | 7.7/19.1 | AGGAGGattaagt**ATG** |  | G1, ORF088 | 161/161 (100) | 161 | 4.0E-110 | YP_241045.1 |
| 23 | 9413 | 9006 | 135 | 5.2/16.5 | AGGAGGaaaaata**ATG** |  | G1, ORF109 | 135/135 (100) | 135 | 3.0E-93 | YP_241046.1 |
| 24 | 9844 | 9413 | 143 | 4.5/17.3 | AGGAGGgataaataata**ATG** |  | G1, ORF103 | 143/143 (100) | 143 | 6.0E-95 | YP_241047.1 |
| 25 | 10038 | 9847 | 63 | 9.9/7.9 | AGGAGGactcaca**ATG** |  | G1, ORF224 | 63/63 (100) | 63 | 7.0E-36 | YP_241048.1 |
| 26 | 10520 | 10035 | 161 | 9.4/18.3 | AGGAGGaagaag**ATG** |  | Sb-1, ORF008 | 161/161 (100) | 161 | 2.0E-109 | AEJ79651.1 |
| 27 | 10944 | 10513 | 143 | 4.2/16.7 | AGGAGAgttaaa**ATG** |  | K, ORF002 | 143/143 (100) | 143 | 2.0E-97 | YP_024433.1 |
| 28 | 11500 | 10958 | 180 | 9.2/21.5 | AGTAGGtaattataaa**ATG** |  | G1, ORF073 | 180/180 (100) | 180 | 6.0E-125 | YP_241051.1 |
| 29 | 12000 | 11512 | 162 | 9.6/19.5 | AGGAGAaatatt**ATG** |  | K, ORF004 | 162/162 (100) | 162 | 1.0E-116 | YP_024435.1 |
| 30 | 12411 | 12013 | 132 | 8.8/16.1 | TGGAGGttaagaa**ATG** |  | K, ORF005 | 132/132 (100) | 132 | 2.0E-88 | YP_024436.1 |
| 31 | 13115 | 12408 | 235 | 4.9/27.7 | AGGAGGagattag**ATG** |  | G1, ORF051 | 235/235 (100) | 235 | 1.0E-170 | YP_241054.1 |
| 32 | 13766 | 13215 | 183 | 4.5/21.1 | AGGAGAtttaaatg**ATG** |  | K, ORF007 | 183/184 (99) | 184 | 2.0E-128 | YP_024438.1 |
| 33 | 14102 | 13785 | 105 | 6.3/11.8 | AGGAGAtaaaattt**GTG** | Major tail protein | G1, ORF138 | 105/105 (100) | 105 | 5.0E-67 | YP_241056.1 |
| 34 | 15636 | 15088 | 182 | 4.5/22.0 | GGGAGTaatat**ATG** |  | K, ORF008 | 182/182 (100) | 182 | 1.0E-123 | YP_024439.1 |
| 35 | 15858 | 15640 | 72 | 4.4/8.4 | AGGAGAggttatgtaa**ATG** |  | G1, ORF201 | 72/72 (100) | 72 | 3.0E-43 | YP_241058.1 |
| 36 | 16053 | 15859 | 64 | 4.6/7.6 | CGGAGGtctata**ATG** |  | G1, ORF218 | 64/64 (100) | 64 | 1.0E-37 | YP_241059.1 |
| 37 | 16780 | 16043 | 245 | 6.2/28.7 | AGGAGGatgttata**ATG** |  | K, ORF009 | 245/245 (100) | 245 | 5.0E-174 | YP_024440.1 |
| 38 | 16947 | 16843 | 34 | 4.7/4.1 | AGGAGGattgct**ATG** |  | G1, ORF437 | 34/34 (100) | 34 | 3.0E-14 | YP_241061.1 |
| 39 | 17198 | 16959 | 79 | 4.7/9.3 | AGGATGagtaat**ATG** |  | A5W, ORF020 | 79/82 (96) | 82 | 2.0E-50 | ACB89011.1 |
| 40 | 17589 | 17200 | 129 | 5.0/15.2 | AGGAGAtgactaatt**ATG** |  | K, ORF010 | 129/129 (100) | 129 | 8.0E-89 | YP_024441.1 |
| 41 | 17861 | 17688 | 57 | 5.1/6.8 | AGGAGGaataaaccct**ATG** |  | G1, ORF245 | 57/57 (100) | 57 | 4.0E-34 | YP_241064.1 |
| 42 | 18384 | 17902 | 160 | 4.5/18.9 | AGGAGGttggtat**ATG** |  | K, ORF011 | 160/160 (100) | 160 | 1.0E-109 | YP_024442.1 |
| 43 | 18976 | 18434 | 180 | 4.8/20.4 | AGGAGAggttaagta**ATG** |  | K, ORF012 | 180/180 (100) | 180 | 7.0E-123 | YP_024443.1 |
| 44 | 19509 | 18976 | 177 | 4.3/20.7 | AGGAGAttaaataaatATG |  | K, ORF013 | 177/177 (100) | 177 | 2.0E-123 | YP_024444.1 |
| 45 | 19676 | 19512 | 54 | 9.5/6.3 | AGGAGTtagagtaact**ATG** |  | G1, ORF256 | 54/54 (100) | 54 | 3.0E-29 | YP_241068.1 |
| 46 | 19954 | 19679 | 91 | 5.0/10.9 | CGGAGGtaggttgta**ATG** |  | G1, ORF163 | 91/91 (100) | 91 | 6.0E-53 | YP_241069.1 |
| 47 | 20799 | 19954 | 281 | 9.2/31.7 | AGGAGGaaaatca**ATG** |  | K, 0RF014 | 281/281 (100) | 281 | 0 | YP_024445.1 |
| 48 | 21929 | 20811 | 372 | 4.7/42.2 | AGGAGAatgataaat**ATG** |  | G1, ORF024 | 372/372 (100) | 372 | 0 | YP_241071.1 |
| 49 | 22409 | 22083 | 108 | 4.7/13.0 | CGGAGAtagtactc**GTG** |  | G1, ORF134 | 108/108 (100) | 108 | 7.0E-73 | YP_241072.1 |
| 50 | 22818 | 22402 | 138 | 5.1/16.0 | AGGAGGgtattac**ATG** |  | K, ORF016 | 138/138 (100) | 138 | 6.0E-95 | YP_024447.1 |
| 51 | 23253 | 22951 | 100 | 4.8/11.3 | AGGAGAtatagaata**ATG** | DNA binding protein | K, ORF017 | 100/100 (100) | 100 | 6.0E-64 | YP_024448.1 |
| 52 | 23441 | 23253 | 62 | 4.3/7.3 | AGGAAGttagaa**ATG** |  | G1, ORF228 | 62/62 (100) | 62 | 1.0E-34 | YP_241075.1 |
| 53 | 23646 | 23485 | 53 | 4.6/6.4 | AGGAGGttgccta**ATG** |  | G1, ORF259 | 53/53 (100) | 53 | 3.0E-29 | YP_241076.1 |
| 54 | 25694 | 23646 | 682 | 6.3/79.8 | AGGAGTgattttaa**ATG** |  | K, ORF018 | 682/682 (100) | 682 | 0 | YP_024449.1 |
| 55 | 26035 | 25772 | 87 | 5.6/10.1 | AGGAGGtttataa**ATG** |  | 676Z, ORF038 | 86/87 (99) | 87 | 8.0E-55 | AFN38278.1 |
| 56 | 26225 | 26052 | 57 | 6.6/6.7 | AGAGGGtaggtaata**TTG** |  | JD007, ORF103 | 57/57 (100) | 57 | 70E-32 | YP_007112812.1 |
| 57 | 26810 | 26232 | 192 | 8.5/21.4 | AGGGGAacaag**ATG** |  | K, ORF019 | 192/192 (100) | 192 | 1.0E-129 | YP_024450.1 |
| 58 | 27429 | 26803 | 208 | 4.6/23.8 | AGGAGAtactatag**ATG** |  | K, ORF020 | 208/208 (100) | 208 | 3.0E-150 | YP_024451.1 |
| 59 | 28318 | 27422 | 298 | 5.4/35.0 | AGGAGGataatta**ATG** | DNA ligase | K, ORF021 | 298/298 (100) | 298 | 0 | YP_024452.1 |
| 60 | 28542 | 28318 | 74 | 8.2/8.2 | AGGAGGattttttt**ATG** |  | JD007, ORF107 | 74/74 (100) | 74 | 5.0E-40 | YP_007112808.1 |
| 61 | 29351 | 28611 | 246 | 5.2/28.6 | AGGGGAactttaaaat**ATG** |  | K, ORF022 | 246/246 (100) | 246 | 0 | YP_024453.1 |
| 62 | 30017 | 29403 | 204 | 4.0/23.0 | AGGAGAaaatatt**ATG** |  | K ,ORF023 | 204/204 (1000 | 204 | 6.0E-144 | YP_024454.1 |
| 63 | 30458 | 30033 | 141 | 6.7/15.8 | TGGAGGattataagt**ATG** | Ribonuclease | K, ORF024 | 141/141 (100) | 141 | 3.00E-94 | YP_024455.1 |
| 64 | 30639 | 30448 | 63 | 5.7/7.5 | TGGAGGtatttgtttt**ATG** |  | G1, ORF222 | 63/63 (100) | 63 | 3.00E-37 | YP_241086.1 |
| 65 | 31303 | 30662 | 213 | 4.0/24.6 | TGGAGGaagacg**ATG** |  | K, ORF025 | 213/213 (100) | 213 | 2.0E-143 | YP_024456.1 |
| 66 | 31523 | 31293 | 76 | 8.0/8.8 | AGGAGAaataatt**ATG** | Transcriptional regulator | G1, ORF187 | 76/76 (100) | 76 | 1.00E-46 | YP_241088.1 |
| 67 | 31753 | 31526 | 75 | 10.0/9.2 | AGGAGGataatga**ATG** |  | G1, ORF190 | 75/75 (100) | 75 | 7.00E-45 | YP_241089.1 |
| 68 | 32555 | 31863 | 230 | 5.0/24.8 | AGGAGTtttaaattt**ATG** |  | K, ORF026 | 230/230 (100) | 230 | 6.0E-167 | YP_024457.1 |
| 69 | 33377 | 32742 | 211 | 9.2/24.8 | AGGAGGagatatt**ATG** |  | K, ORF027 | 211/211 (100) | 211 | 2.0E-151 | YP_024458.1 |
| 70 | 34235 | 33444 | 263 | 8.9/29.3 | AGGAGAaatgta**ATG** |  | K, ORF028 | 263/263 (100) | 263 | 0 | YP_024459.1 |
| 71 | 34543 | 34235 | 102 | 8.8/12.1 | AGGAGGaattac**ATG** |  | K, ORF029 | 102/102 (100) | 102 | 7.0E-65 | YP_024460.1 |
| 72 | 35285 | 34656 | 209 | 9.7/23.1 | AGTCTGtcaatca**ATG** | Amidase | G1, ORF060 | 209/209 (100) | 209 | 5.0E-154 | YP_241094.1 |
| 73 | 36056 | 35556 | 166 | 9.3/19.2 | AGGAGAtgttatt**ATG** | Endonuclease | K, ORF031 | 166/166 (100) | 166 | 3.0E-116 | YP_024462.1 |
| 74 | 37019 | 36216 | 267 | 9.5/29.8 | AGGAGGaagttaagta**ATG** | CHAP domain | G1, ORF042 | 267/267 (100) | 267 | 0.0E+00 | YP_241096.1 |
| 75 | 37522 | 37019 | 167 | 4.1/18.1 | AGGTCGgtttttta**ATG** | Putative holin | K, ORF033 | 167/167 (100) | 167 | 7.0E-116 | YP_024463.1 |
| 76 | 37792 | 37607 | 61 | 5.0/7.1 | AGGAGAgatacaa**ATG** |  | G1, ORF233 | 61/61 (100) | 61 | 1.0E-33 | YP_241098.1 |
| 77 | 39557 | 39339 | 72 | 9.2/8.7 | AGGAATtgattaatt**ATG** |  | G1, ORF200 | 72/72 (100) | 72 | 4.0E-45 | YP_241099.1 |
| 78 | 40244 | 40035 | 69 | 5.7/8.1 | AGGAGAgattgtag**ATG** |  | G1, ORF207 | 69/69 (100) | 69 | 2.0E-42 | YP_241100.1 |
| 79 | 40589 | 40257 | 110 | 5.0/12.5 | AGGTGAttctag**TTG** |  | G1, ORF209 | 110/110 (100) | 110 | 3.0E-69 | YP_241101.1 |
| 80 | 40928 | 40602 | 108 | 5.6/13.0 | AGGAGGttaccact**TTG** | Membrane protein | GH15, ORF077 | 108/108 (100) | 108 | 6.0E-71 | YP_007002200.1 |
| 81 | 41227 | 40961 | 88 | 9.4/10.1 | AGGTGTttaaca**ATG** |  | G1, ORF169 | 88/88 (100) | 88 | 4.0E-50 | YP_241102.1 |
| 82 | 41368 | 41754 | 128 | 9.1/14.8 | AGGAGGttaaagtgg**TTG** |  | G1, ORF168 | 128/128 (100) | 128 | 7.0E-82 | YP_241103.1 |
| 83 | 41732 | 42010 | 92 | 9.7/10.6 | AGGAAGattaca**ATG** |  | G1, ORF161 | 92/92 (100) | 92 | 2.0E-60 | YP_241104.1 |
| 84 | 42007 | 42417 | 136 | 4.4/15.6 | AGGTGAaatgga**TTG** |  | G1, ORF133 | 136/136 (100) | 136 | 2.0E-91 | YP_241105.1 |
| 85 | 42432 | 42629 | 65 | 9.6/7.7 | AGGAGAaagatataa**ATG** | Small subunit of the terminase | Twort, ORF151 | 64/79 (81) | 79 | 4.0E-38 | YP_238726.1 |
| 86 | 42923 | 43894 | 323 | 9.1/38.6 | AGGTGAaaacag**TTG** |  | MSA6, ORF058 | 323/323 (100) | 323 | 0. | AFN38731.1 |
| 87 | 44035 | 45582 | 515 | 5.8/59.7 | AGGTCTtagtgaa**ATG** | Large subunit of the terminase | K, ORF035 | 515/605 (85) | 605 | 0 | YP_024465.1 |
| 88 | 45575 | 46396 | 273 | 5.1/30.6 | AGAAGGaaataaacaattctactttg**ATG** |  | ISP, ORF002 | 273/273 (100) | 273 | 0 | CCA65732.1 |
| 89 | 46383 | 46556 | 57 | 9.4/6.7 | AGGTGAttata**GTG** |  | JD007, ORF129 | 57/57 (100) | 57 | 9.00E-30 | YP_007112784.1 |
| 90 | 46553 | 47032 | 159 | 4.8/18.5 | AGGAAGaaataa**ATG** |  | K, ORF037 | 159/159 (100) | 159 | 2.0E-109 | YP_024467.1 |
| 91 | 47125 | 48243 | 372 | 4.1/40.8 | ATGAGGtaatcataaccataataacggtt**ATG** |  | K, ORF038 | 370/397 (93) | 397 | 0 | YP_024468.1 |
| 92 | 48320 | 48670 | 116 | 9.3/13.1 | CGGTGGtgaaaact**TTG** |  | G1, ORF120 | 116/166 (100) | 116 | 1.00E-73 | YP_240898.1 |
| 93 | 48688 | 49059 | 123 | 5.7/14.5 | AGACGGtgaatagg**TTG** |  | K, ORF040 | 123/123 (100) | 123 | 9.00E-83 | YP_024470.1 |
| 94 | 49063 | 50754 | 563 | 6.1/64.1 | AGGTGActagtaa**TTG** | Portal protein | K, ORF041 | 563/563 (100) | 563 | 0 | YP_024471.1 |
| 95 | 50948 | 51721 | 257 | 4.9/28.6 | TGGAGGtgtagacacct**TTG** | Prohead protease | K, ORF042 | 257/257 (100) | 257 | 0 | YP_024472.1 |
| 96 | 51740 | 52696 | 318 | 4.4/36.0 | AGGAGAatacattct**ATG** |  | G1, ORF029 | 318/318 (100) | 318 | 0 | YP_240902.1 |
| 97 | 52812 | 54203 | 463 | 5.1/51.2 | AGGTGAtaaatttat**ATG** | Major capsid protein | K ,ORF044 | 463/463 (100) | 463 | 0 | YP_024474.1 |
| 98 | 54295 | 54591 | 98 | 9.4/11.3 | AGGGATttaataaat**ATG** |  | G1, ORF151 | 98/98 (100) | 98 | 1.00E-59 | YP_240904.1 |
| 99 | 54604 | 55512 | 302 | 5.1/34.2 | AGGGTGaattaa**ATG** |  | K ,ORF045 | 302/302 (100) | 302 | 0 | YP_024475.1 |
| 100 | 55526 | 56404 | 292 | 5.6/33.8 | AGGAGGgttagaaa**ATG** | Capsid protein | K ,ORF046 | 292/292 (100) | 292 | 0 | YP_024476.1 |
| 101 | 56404 | 57024 | 206 | 10.3/23.8 | CGGAGGtgcatttaaata**ATG** |  | K, ORF047 | 206/206 (100) | 206 | 2.0E-148 | YP_024477.1 |
| 102 | 57043 | 57879 | 278 | 4.7/31.8 | AGGAGGgttagtattaa**ATG** |  | K ,ORF048 | 278/278 (100) | 278 | 0 | YP_024478.1 |
| 103 | 57881 | 58096 | 71 | 8.0/8.3 | TGGAAGtagg**ATG** |  | G1, ORF202 | 71/71 (100) | 71 | 2.0E-45 | YP_240909.1 |
| 104 | 58123 | 59886 | 587 | 4.9/64.5 | AGGAGAattaaat**ATG** | Tail sheath protein | K ,ORF049 | 586/587 (99) | 587 | 0 | YP_024479.1 |
| 105 | 59959 | 60387 | 142 | 5.4/16.0 | AGGAGAgtgaatacag**ATG** | Structural protein | K ,ORF050 | 142/142 (100) | 142 | 1.0E-99 | YP_024480.1 |
| 106 | 60484 | 60624 | 46 | 10.7/5.4 | AGGAGAtgtactag**ATG** |  | G1, ORF293 | 43/43 (100) | 43 | 4.0E-23 | YP_240912.1 |
| 107 | 60667 | 61125 | 152 | 9.6/18.1 | AGGAGAtgggtat**ATG** |  | K, ORF051 | 152/152 (100) | 152 | 1.0E-107 | YP_024481.1 |
| 108 | 61138 | 61332 | 64 | 9.5/7.1 | AGGAGGtattata**ATG** |  | G1, ORF215 | 64/64 (100) | 64 | 2.0E-34 | YP_240914.1 |
| 109 | 61414 | 61725 | 103 | 5.9/12.3 | AGGAGAagatataaa**ATG** |  | K ,ORF052 | 103/103 (100) | 103 | 8.0E-66 | YP_024482.1 |
| 110 | 61928 | 62314 | 128 | 4.7/15.3 | AGATGTtagtaaa**ATG** |  | K ,ORF053 | 128/152 (84) | 152 | 2.0E-88 | YP_024483.1 |
| 111 | 62358 | 62894 | 178 | 4.2/21.0 | AGAACGattgggcggt**ATG** | RNA polymerase | K ,ORF054 | 178/178 (100) | 178 | 1.0E-125 | YP_024484.1 |
| 112 | 62950 | 63093 | 47 | 4.0/5.3 | TGGATGgtgaataatg**ATG** |  | GH15, ORF107 | 46/1352 (0.03) | 1352 | 2.0E-21 | YP_007002230.1 |
| 113 | 63132 | 67004 | 1290 | 9.2/137.0 | AGAACGca**ATG** | Tail measure protein | G1, ORF001 | 1290/1352 (95) | 1352 | 0 | YP_240918.1 |
| 114 | 67083 | 69509 | 808 | 6.3/91.2 | AGGAAGtatgtgtat**ATG** | Tail endolysin | K, ORF056 | 808/808 (100) | 808 | 0 | YP_024486.1 |
| 115 | 69523 | 70410 | 295 | 4.5/34.6 | AGGAGGatagtct**ATG** |  | K, ORF057 | 295/295 (100) | 295 | 0 | YP_024487.1 |
| 116 | 70410 | 72956 | 848 | 4.8/96.1 | AAGAGGtgtatattta**ATG** |  | G1, ORF004 | 848/848 (100) | 848 | 0 | YP_240923.1 |
| 117 | 73062 | 73853 | 263 | 7.8/29.3 | AGGAGAggataaa**ATG** |  | K, ORF059 | 263/263 (100) | 263 | 0 | YP_024489.1 |
| 118 | 73853 | 74377 | 174 | 4.5/20.0 | TGGAGGtgtatctagcta**ATG** |  | G1, ORF078 | 174/174 (100) | 174 | 3.0E-121 | YP_240925.1 |
| 119 | 74377 | 75081 | 234 | 4.6/26.6 | AGGAGGctaacgtata**ATG** | Baseplate protein | K, ORF061 | 234/234 (100) | 234 | 5.0E-171 | YP_024491.1 |
| 120 | 75096 | 76142 | 348 | 4.7/39.2 | AGGATTaaatt**ATG** | Tail protein | K, ORF062 | 348/348 | 348 | 0 | YP_024492.1 |
| 121 | 76163 | 79222 | 1019 | 5.0/116.3 | AGGTGAaacttaagtc**GTG** |  | G1, ORF003 | 1019/1019 (100) | 1019 | 0 | YP_240928.1 |
| 122 | 79333 | 79854 | 173 | 5.3/19.2 | AGGAATtaaaaaat**ATG** | Structural protein | K, ORF064 | 173/173 (100) | 173 | 3.0E-122 | YP_024494.1 |
| 123 | 79875 | 83333 | 1152 | 5.1/129.1 | GGGAGAtaattctaa**ATG** | Adsorption associated protein | K, ORF065 | 1152/1152 (100) | 1152 | 0 | YP_024495.1 |
| 124 | 83382 | 83540 | 52 | 8.0/6.2 | AGGAGAgatttat**ATG** |  | G1, ORF262 | 52/52 (100) | 52 | 8.0E-27 | YP_240931.1 |
| 125 | 83541 | 85463 | 640 | 6.3/72.6 | AGGAGAaaaatag**ATG** |  | G1, ORF009 | 639/640 (99) | 640 | 0 | YP_240932.1 |
| 126 | 85486 | 85860 | 124 | 4.7/14.6 | AGGTGGaataaaaaaact**ATG** |  | K, ORF067 | 124/124 (100) | 124 | 10E-83 | YP_024497.1 |
| 127 | 85867 | 87243 | 458 | 5.9/50.4 | AGGGGTaattataa**ATG** |  | G1, ORF017 | 458/458 (100) | 458 | 0 | YP_240934.1 |
| 128 | 87334 | 89082 | 582 | 5.6/67.2 | AGGAGAttaaa**ATG** | Helicase | K, ORF069 | 582/582 (100) | 582 | 0 | YP_024499.1 |
| 129 | 89094 | 90707 | 537 | 8.0/63.2 | AGGAGGgtaagag**ATG** |  | K ,ORF070 | 537/537 (100) | 537 | 0 | YP_024500.1 |
| 130 | 90700 | 92142 | 480 | 5.5/54.6 | AAGAGGttaataact**ATG** | DNA helicase | K, ORF071 | 480/480 (100) | 480 | 0 | YP_024501.1 |
| 131 | 92221 | 93258 | 345 | 4.7/40.1 | AGGAGAgattaata**ATG** | Exonuclease | K, ORF072 | 345/345 (100) | 345 | 0 | YP_024502.1 |
| 132 | 93258 | 93635 | 125 | 5.2/14.9 | AGGAGGttttata**ATG** |  | K, ORF073 | 125/125 (100) | 125 | 2.0E-85 | YP_024503.1 |
| 133 | 93635 | 95554 | 639 | 5.1/73.4 | AGGGGAtaagta**ATG** | Exonuclease | K, ORF074 | 639/639 (100) | 639 | 0 | YP_024504.1 |
| 134 | 95554 | 96150 | 198 | 6.3/23.2 | TGGAGGaaaaata**ATG** |  | K, ORF075 | 198/198 (100) | 198 | 5.0E-142 | YP_024505.1 |
| 135 | 96165 | 97232 | 355 | 8.5/40.9 | AGGAAGgatatt**ATG** | DNA primase | K, ORF076 | 355/355 (100) | 355 | 0 | YP_024506.1 |
| 136 | 97298 | 97636 | 112 | 4.2/13.0 | AGGAGAaaaaaata**ATG** |  | G1, ORF127 | 112/112 (100) | 112 | 1.0E-71 | YP_240943.1 |
| 137 | 97636 | 98088 | 150 | 4.8/17.1 | AGGAGAacaagaata**ATG** |  | Sb-1, ORF120 | 150/150 (100) | 150 | 4.0E-100 | AEJ79755.1 |
| 138 | 98075 | 98683 | 202 | 5.5/23.6 | AGTTGaagaaaaATG | Resolvase | G1, ORF064 | 202/202 (100) | 202 | 1.0E-147 | YP_240945.1 |
| 139 | 98661 | 99092 | 143 | 9.8/16.2 | ATGAATtgatta**ATG** |  | A5W, ORF109 | 143/143 (100) | 143 | 4.0E-97 | ACB89102.1 |
| 140 | 99107 | 101221 | 704 | 5.6/80.1 | AGGATGagagatat**ATG** |  | G1, ORF006 | 702/704 (99) | 704 | 0 | YP_240947.1 |
| 141 | 101235 | 102284 | 349 | 4.7/40.5 | AGGAAAtagat**ATG** |  | K, ORF081 | 349/349 (100) | 349 | 0 | YP_024511.1 |
| 142 | 102302 | 102631 | 109 | 4.5/12.4 | AGGAGAaaagatatt**ATG** |  | K, ORF082 | 109/109 (100) | 109 | 2.0E-72 | YP_024512.1 |
| 143 | 102615 | 102935 | 106 | 4.8/12.1 | AGGATGattcagaag**ATG** |  | K, ORF083 | 106/106 (100) | 106 | 6.0E-69 | YP_024513.1 |
| 144 | 103178 | 103738 | 186 | 6.5/22.1 | AGAAGAtatatc**ATG** |  | K, ORF084 | 186/198 (94) | 198 | 1.0E-131 | YP_024514.1 |
| 145 | 103748 | 104053 | 101 | 5.8/11.9 | AGGTGAttat**ATG** | Integration host factor | K, ORF085 | 101/101 (100) | 101 | 4.0E-66 | YP_024515.1 |
| 146 | 104129 | 105001 | 290 | 5.9/33.2 | AGGAGAggaattaa**ATG** | DNA polymerase | G1, ORF035 | 290/290 (100) | 290 | 0 | YP_240953.1 |
| 147 | 105167 | 105679 | 170 | 9.7/20.3 | AGGTGAgacata**GTG** |  | G1, ORF081 | 170/170 (100) | 170 | 3.0E-118 | YP_240954.1 |
| 148 | 105815 | 107158 | 447 | 5.3/52.8 | ATTAAGttctta**ATG** | DNA polymerase | K, ORF86 | 445/1072 (41) | 1072 | 0 | YP_024516.1 |
| 149 | 107426 | 108133 | 235 | 9.6/27.5 | AGGAGAgttat**ATG** | HNH endonuclease | K, ORF089 | 235/269 (87) | 269 | 4.0E-169 | YP_024518.1 |
| 150 | 108367 | 109227 | 286 | 4.9/32.9 | AGGGTCcgactatagcgccctagag**ATG** | DNA polymerase | G1, ORF037 | 286/286 (100) | 286 | 0 | YP_240958.1 |
| 151 | 109296 | 109538 | 80 | 4.2/9.1 | AGGAGGaaaaaga**GTG** |  | G1, ORF181 | 80/80 (100) | 80 | 7.0E-50 | YP_240959.1 |
| 152 | 109555 | 110037 | 160 | 5.5/18.9 | AGGAGGaactgataa**ATG** |  | G1, ORF091 | 160/160 (100) | 160 | 1.0E-114 | YP_024519.1 |
| 153 | 110124 | 111395 | 423 | 4.6/46.9 | AGGAGAaaagataatt**ATG** |  | K, ORF092 | 423/423 (100) | 423 | 0 | YP_024520.1 |
| 154 | 111455 | 111679 | 74 | 6.1/7.9 | AGGAGGatattaa**ATG** | Recombinase | K, ORF093 | 71/418 (17) | 418 | 1.0E-39 | YP_024521.1 |
| 155 | 112024 | 112992 | 322 | 9.3/38.3 | TGGAGAgtagtataat**ATG** | Endonuclease | SA11, ORF083 | 282/322 (88) | 322 | 0 | YP_007005558.1 |
| 156 | 113140 | 114087 | 315 | 5.1/35.7 | AGCAGAtaacaatcgt**ATG** | Recombinase | K, ORF093 | 315/418 (75) | 418 | 0 | YP_024521.1 |
| 157 | 114091 | 114444 | 117 | 5.1/13.4 | TGGAGAttaagctt**ATG** |  | G1, ORF121 | 117/117 (100) | 117 | 5.0E-79 | YP_240963.1 |
| 158 | 114431 | 115093 | 220 | 5.3/26.6 | AGGGGGctaatcctt**ATG** |  | K, ORF094 | 220/220 (100) | 220 | 9.0E-156 | YP_024522.1 |
| 159 | 115221 | 115853 | 210 | 4.7/23.2 | TGGAGAttaagctt**ATG** |  | K ,ORF095 | 210/210 (100) | 210 | 2.0E-149 | YP_024523.1 |
| 160 | 115876 | 116388 | 170 | 4.3/17.9 | AGGATGgtaaat**TTG** | Major tail protein | G1, ORF080 | 170/170 (100) | 170 | 1.0E-113 | YP_240966.1 |
| 161 | 116403 | 116630 | 75 | 4.4/7.8 | AGGAGGacaataaaga**ATG** | Major tail protein | G1, ORF189 | 75/75 (100) | 75 | 8.0E-45 | YP_240967.1 |
| 162 | 116726 | 116986 | 86 | 5.6/10.3 | AGGAGGggaaagta**ATG** |  | G1, ORF174 | 86/86 (100) | 86 | 1.0E-54 | YP_240968.1 |
| 163 | 116990 | 117745 | 251 | 4.4/29.2 | AGTAGGtgagtagggt**ATG** |  | K, ORF097 | 251/251 (100) | 251 | 6.0E-180 | YP_024525.1 |
| 164 | 117738 | 118988 | 416 | 5.7/47.5 | AGGAAGcagtt**ATG** | DNA polymerase | K, ORF098 | 416/416 (100) | 416 | 0 | YP_024526.1 |
| 165 | 119002 | 119370 | 122 | 5.6/14.0 | AGGATGgttaataa**ATG** |  | K, ORF099 | 122/122 (100) | 122 | 2.0E-80 | YP_024527.1 |
| 166 | 119357 | 119668 | 103 | 4.6/12.0 | AGGAGGataata**ATG** |  | K, ORF100 | 103/103 (100) | 103 | 6.0E-68 | YP_024528.1 |
| 167 | 119732 | 120268 | 178 | 6.9/20.8 | AGGTGActtaaaa**ATG** |  | G1, ORF075 | 178/178 (100) | 178 | 5.0E-127 | YP_240973.1 |
| 168 | 120261 | 121028 | 255 | 9.6/30.1 | AGGAGAtaggttaa**ATG** |  | G1, ORF045 | 255/255 (100) | 255 | 0 | YP_240974.1 |
| 169 | 121006 | 121398 | 130 | 9.6/15.1 | AAGAGTaatta**ATG** |  | SA5, ORF080 | 130/130 (100) | 1630 | 2.0E-88 | AFV80741.1 |
| 170 | 121451 | 122314 | 287 | 5.5/32.4 | AGGAAAgataaata**ATG** |  | G1, ORF036 | 287/287 (100) | 287 | 0 | YP_240976.1 |
| 171 | 122686 | 123417 | 243 | 5.2/28.4 | AGGAGAgctatata**ATG** |  | K, ORF103 | 243/243 (100) | 243 | 1.0E-172 | YP_024531.1 |
| 172 | 123435 | 123893 | 152 | 4.8/17.8 | GTGAGGtatagagta**ATG** |  | G1, ORF094 | 152/152 (100) | 152 | 5.0E-105 | YP_240978.1 |
| 173 | 123958 | 124401 | 147 | 6.0/17.5 | AGGAGCtaacaatt**ATG** |  | K, ORF105 | 147/147 (100) | 147 | 2.0E-98 | YP_024533.1 |
| 174 | 124418 | 125122 | 234 | 4.6/27.4 | AAGAGGttataat**ATG** |  | K ,ORF106 | 234/234 (100) | 234 | 5.0E-168 | YP_024534.1 |
| 175 | 125184 | 125582 | 132 | 8.9/15.4 | TAGAGGtgttaatt**ATG** |  | K, ORF107 | 132/132 (100) | 132 | 5.0E-90 | YP_024535.1 |
| 176 | 125729 | 125971 | 80 | 9.3/9.4 | AGGAGAgattaact**ATG** |  | G1, ORF182 | 80/80 (100) | 80 | 3.0E-48 | YP_240982.1 |
| 177 | 125976 | 126140 | 54 | 6.2/6.3 | AGGAGAtaggaca**ATG** |  | G1, ORF252 | 54/54 (100) | 54 | 2.0E-29 | YP_240983.1 |
| 178 | 126127 | 126306 | 59 | 9.3/7.1 | ATTAGAttctattatgg**TTG** |  | A5W, ORF146 | 59/59 (100) | 59 | 2.0E-33 | ACB89139.1 |
| 179 | 126342 | 126518 | 58 | 4.6/7.0 | AGGAGGagagatatt**ATG** |  | G1, ORF240 | 58/58 (100) | 58 | 2.0E-32 | YP_240984.1 |
| 180 | 126924 | 127043 | 39 | 8.0/4.9 | TGGAAAaaccaa**TTG** |  | G1, ORF076 | 38/177 (22) | 177 | 2.0E-16 | YP_240985.1 |
| 181 | 127058 | 127306 | 82 | 4.7/9.1 | AGGTGGaaagca**ATG** |  | ISP, ORF92 | 82/82 (100) | 82 | 7.0E-45 | CCA65823.1 |
| 182 | 127318 | 127494 | 58 | 9.9/7.0 | AGGAGAtttact**ATG** |  | G1, ORF241 | 58/58 (100) | 58 | 6.0E-31 | YP_240986.1 |
| 183 | 127487 | 127783 | 98 | 6.6/11.3 | AGGAGAaaaagaa**ATG** |  | G1, ORF152 | 98/98 (100) | 98 | 3.0E-63 | YP_240987.1 |
| 184 | 127831 | 128013 | 60 | 8.2/7.2 | AAGAGGaatgattatt**ATG** |  | G1, ORF219 | 59/60 (98) | 60 | 2.0E-32 | YP_240988.1 |
| 185 | 128026 | 128394 | 122 | 4.4/14.2 | AGGAGGttgtatag**ATG** |  | G1, ORF119 | 122/122 (100) | 122 | 1.0E-81 | YP_240989.1 |
| 186 | 128407 | 128754 | 115 | 4.6/13.0 | AGGAGGaaataga**ATG** |  | G1, ORF124 | 115/115 (100) | 115 | 2.0E-76 | YP_240990.1 |
| 187 | 128754 | 129032 | 92 | 4.3/10.2 | TGGAAGactggaactgggatta**ATG** |  | G1, ORF162 | 92/92 (100) | 92 | 5.0E-56 | YP_240991.1 |
| 188 | 129102 | 129407 | 101 | 9.2/12.1 | AGGAGAtgattact**ATG** |  | G1, ORF140 | 101/101 (100) | 101 | 7.0E-67 | YP_240992.1 |
| 189 | 129422 | 129772 | 116 | 10.0/13.7 | AGGAGGtggaaag**ATG** |  | G1, ORF122 | 116/116 (100) | 116 | 4.0E-76 | YP_240993.1 |
| 190 | 129772 | 130374 | 200 | 9.7/23.4 | AGGAGGaaaaata**ATG** |  | G1, ORF065 | 200/200 (100) | 200 | 2.0E-144 | YP_240994.1 |
| 191 | 130388 | 130567 | 59 | 9.0/7.3 | AGGTGGttatataa**ATG** |  | G1, ORF237 | 59/59 (100) | 59 | 2.0E-34 | YP_240995.1 |
| 192 | 130793 | 131194 | 133 | 4.9/15.0 | AGGAGTggttgta**ATG** |  | G1, ORF107 | 133/133 (100) | 133 | 2.0E-86 | YP_240996.1 |
| 193 | 131196 | 131456 | 86 | 4.4/10.1 | AGGAGGaaattaaa**ATG** |  | G1, ORF173 | 86/86 (100) | 86 | 4.0E-53 | YP_240997.1 |
| 194 | 131508 | 131795 | 95 | 9.2/10.5 | AGGAGGaaattaat**ATG** |  | G1, ORF157 | 95/95 (100) | 95 | 5.0E-59 | YP_240999.1 |
| 195 | 131806 | 131922 | 38 | 4.8/4.6 | AGGAGGaattct**ATG** | Transcription factor | G1, ORF362 | 38/38 (100) | 38 | 8.0E-15 | YP_241000.1 |
| 196 | 131912 | 132175 | 87 | 10.1/9.9 | AGGAGGaaaaacaaaag**ATG** |  | G1, ORF170 | 87/87 (100) | 87 | 7.0E-53 | YP_241001.1 |
| 197 | 132252 | 132431 | 59 | 10.1/6.4 | AGGAGGttaattt**ATG** |  | G1, ORF236 | 59/59 (100) | 59 | 3.0E-29 | YP_241002.1 |
| 198 | 132446 | 132709 | 87 | 4.8/10.3 | AGGAGGaactataa**ATG** |  | G1, ORF171 | 87/87 (100) | 87 | 1.0E-55 | YP_241003.1 |
| 199 | 132712 | 133029 | 105 | 5.0/12.0 | AGGAGGaaaattaatt**ATG** |  | G1, ORF137 | 105/105 (100) | 105 | 7.0E-68 | YP_241004.1 |
| 200 | 133030 | 133128 | 32 | 8.2/3.6 | AGGAGGaaaaataa**GTG** |  | G1, ORF055 | 32/226 (14) | 226 | 9.0E-13 | YP_241006.1 |
| 201 | 133396 | 133710 | 104 | 5.1/11.6 | ATGAATattattca**ATG** |  | G1, ORF055 | 104/226 (46) | 226 | 4.0E-66 | YP_241006.1 |
| 202 | 133799 | 133957 | 52 | 5.8/5.7 | AGGAGGtaatata**ATG** |  | G1, ORF263 | 52/52 (100) | 52 | 3.0E-23 | YP_241007.1 |
| 203 | 133992 | 134192 | 66 | 5.1/7.6 | AGGAGAggtaaat**ATG** |  | G1, ORF211 | 66/66 (100) | 66 | 4.0E-41 | YP_241008.1 |
| 204 | 134193 | 134483 | 96 | 8.9/11.1 | CGGAGGggttataa**ATG** |  | G1, ORF155 | 96/96 (100) | 155 | 5.0E-59 | YP_241009.1 |
| 205 | 134575 | 134883 | 102 | 5.5/12.0 | AGGAGAtgaacaatt**ATG** |  | K, ORF109 | 102/102 (100) | 102 | 2.0E-63 | YP_024537.1 |
| 206 | 134880 | 135788 | 302 | 5.1/35.2 | AGGAGCtatcaacaaa**ATG** |  | K, ORF110 | 302/302 (100) | 302 | 0 | YP_024538.1 |
| 207 | 135806 | 137275 | 489 | 5.3/56.1 | AGGAGAaaaataaatt**ATG** |  | K, ORF111 | 489/489 (100) | 489 | 0 | YP_024539.1 |
| 208 | 137354 | 137599 | 81 | 8.8/10.0 | AGGAGTgaaagaa**ATG** |  | G1, ORF178 | 81/81 (100) | 81 | 2.0E-51 | YP_241013.1 |
| 209 | 137619 | 138011 | 130 | 5.1/15.4 | AGGAGAgaaataaa**ATG** |  | K, ORF113 | 130/130 (100) | 130 | 2.0E-86 | YP_241014.1 |
| 210 | 138013 | 138234 | 73 | 4.5/8.9 | AGGAGAgaaatagc**ATG** |  | G1, ORF194 | 72/73 (99) | 73 | 2.0E-43 | YP_241015.1 |
| 211 | 138300 | 138611 | 103 | 5.1/11.6 | AGGAGAtgaaaat**ATG** |  | G1, ORF142 | 103/103 (100) | 103 | 1.0E-65 | YP_241016.1 |
| 212 | 138614 | 139123 | 169 | 9.3/20.3 | AGGAGActaact**ATG** |  | G1, ORF082 | 169/169 (100) | 169 | 6.0E-118 | YP_241017.1 |
| 213 | 139125 | 139454 | 109 | 5.3/12.6 | AGGAAGtgtttaagtaat**ATG** |  | G1, ORF131 | 109/109 (100) | 109 | 3.0E-73 | YP_241018.1 |
| 214 | 139460 | 139654 | 64 | 7.8/7.8 | GGGAGTaattata**ATG** |  | A5W, ORF179 | 64/64 (100) | 64 | 1.0E-36 | ACB89172.1 |
| 215 | 139678 | 139992 | 104 | 4.1/12.0 | AGGAGGagtaaactatt**ATG** |  | G1, ORF139 | 104/104 (100) | 104 | 2.0E-66 | YP_241019.1 |
| 216 | 139986 | 140174 | 62 | 4.3/7.3 | AGCAGGgttagattacttaaatattaa**ATG** |  | A5W, ORF181 | 62/62 (100) | 62 | 3.0E-35 | ACB89174.1 |
| 217 | 140211 | 140312 | 33 | 5.1/3.7 | AGGAGGaaacat**ATG** |  | G1, ORF445 | 33/33 (100) | 33 | 2.0E-13 | YP_241021.1 |
